# Determination of Particle Size Distributions of Bulk Samples Using Micro-Computed Tomography and Artificial Intelligence

**DOI:** 10.3390/ma16031002

**Published:** 2023-01-21

**Authors:** Stefan Höving, Laura Neuendorf, Timo Betting, Norbert Kockmann

**Affiliations:** Laboratory of Equipment Design, Department of Biochemical and Chemical Engineering, TU Dortmund University, Emil-Figge-Straße 68, 44227 Dortmund, Germany

**Keywords:** determination of particle size distributions, three dimensional particle analysis in bulk, micro-computed tomography, Mask–RCNN, Wadell sphericity

## Abstract

The knowledge of product particle size distribution (PSD) in crystallization processes is of high interest for the pharmaceutical and fine chemical industries, as well as in research and development. Not only can the efficiency of crystallization/production processes and product quality be increased but also new equipment can be qualitatively characterized. A large variety of analytical methods for PSDs is available, most of which have underlying assumptions and corresponding errors affecting the measurement of the volume of individual particles. In this work we present a method for the determination of particle volumes in a bulk sample via micro-computed tomography and the application of artificial intelligence. The particle size of bulk samples of sucrose were measured with this method and compared to classical indirect measurement methods. Advantages of the workflow are presented.

## 1. Introduction

The use of computed tomography-generated data in disciplines, such as structural geology, minerals engineering, and soil science, is common [1,2,3]. It allows for the structural analysis of bore and bulk samples. Characteristics, such as porosity [4,5] and air void content [6], and textual information, such as shape [7,8] and permeability [9] of the sample, can be determined. These characteristics yield information about the nature of the respective sample and do not necessarily describe individual particles. Although this is part of recent investigations in spray dried, spherical particles and metal particles [10,11,12,13], non-spherical particles are seldom covered. Samples containing spherical individual particles allow for efficient application of artificial intelligence (AI) methods and post-processing algorithms. When it comes to the study of particle size distribution of individual non-spherical particles in a bulk sample, with the help of artificial intelligence, available literature becomes scarce. However, one contribution using AI algorithms to determine particle mixtures in images of fluid catalytic cracking was investigated by Frei et al. [14]. Gouillart et al. describe how Python libraries can be used to investigate X-ray images [15].

Particle size distributions (PSDs) play a mayor role in industrial crystallization processes. Different PSDs have different mechanical properties. In most cases, large product particles, with narrow PSDs, are preferred, as they bring advantages such as: reduced formation of clumps, easy filtration, easy dosing and storing, and increased drying capability. At the same time, fines can also have benefits. such as increased solubility and compressibility.

On the one hand, production processes in fine chemistry and pharmacy rely on product characterization methods regarding size distributions aiming towards production cost efficiency and product quality [16,17,18]. On the other hand, research and development depend on characterization experiments of new equipment and, therefore, need product particle analysis [19,20].

Different approaches have emerged with their individual technologies, advantages, and drawbacks. The measurement of single particle shape and size properties in high quantity is not trivial, and often representative quantities are measured with necessary assumptions. To allow some sort of comparability, equivalent spherical diameters are frequently used to describe the size of individual particles. An overview of commonly used technology in these fields is given in Table 1. Due to all of the underlying different approximations, it is especially important to note the resulting systematic errors and the transferability of results between different measurement methods. It is important to consider assumptions and approximations made as well as the measurement technique [21]. The relevant techniques have been described in several standard text books addressing particle measurements [20,21,22,23,24,25,26,27].

In this contribution we present a method that aims towards determining the volumes of single particles from a bulk sample without having to resort to measuring a representative quantity, such as the sphere equivalent diameter, and calculating the sphere volume accordingly. For this purpose, we developed a sampler for a micro-computer tomograph (µCT) that allows for the non-invasive, non-destructive measurement of a bulk of particles in the three dimensional space [29]. Bulk samples can be investigated as a whole, determining individual particle features, PSDs, or the porosity of the sample [30,31,32]. Similar data can be obtained using the commercial service offered by Xnovo Technology ApS as a commercial service [Køge, Denmark]. However, here the measurement technique is based on X-ray diffraction.

With the help of artificial intelligence (AI) the images are segmented into background and individual particles for each slice of the 3D scan. A specially self-developed post-processing software reconstructs the segmented slices of the scan to volumes of individual particles in the bulk sample and enables actual determination of the particle’s form and size. Hence, volume and form factors can also be determined.

## 2. Materials and Methods

This section deals with the experimental setup used to generate the presented measurements and results. The workflow, in terms of µCT measurements, reconstruction, application of AI, post-processing, and evaluation, are discussed. Additionally, the investigated particle species are introduced.

### 2.1. Experimental Setup

This subsection is about the µCT used and specifies the particle properties of the analyzed particle species.

#### 2.1.1. µCT

**Figure 1:**
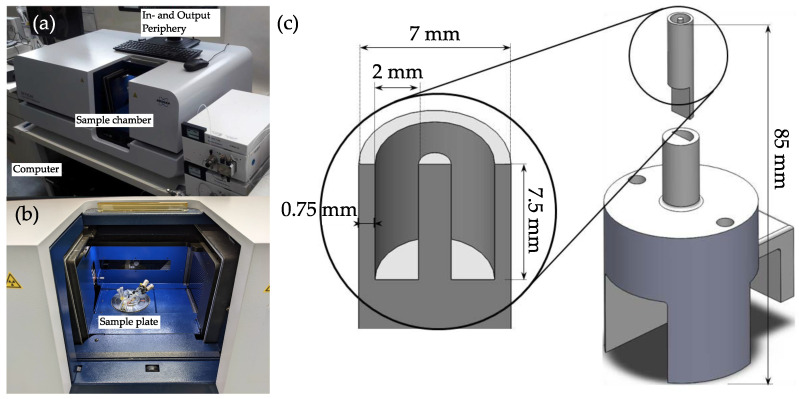
Images of the µCT device (**a**) and the sample chamber (**b**). (**c**) shows the holder for the crystalline sample (top) made from PTFE. The bottom part can be installed to the rotating sample plate and is 3D-printed from PLA.

For data collection, the µCT-model Bruker(R) Skyscan 1275 (RJL Micro Analytic GmbH, Karlsdorf Neuthart, Germany) was used [33]. It is a laboratory-scale µCT, which enables 3D image reconstruction from a rotating object. This allows for non-destructive tomographic analysis of small samples (∅ = 9.6 cm, H = 12 cm) with a spacial resolution of down to 4 µm and a time resolution of 30–150 ms per scan. The sample of interest can be fixed in the middle of the sample chamber, where the sample plate rotates at 0.12° angle steps for data acquisition(cf. Figure 1. Set point parameters for the measurements are given in Table 2.

To evaluate and view the scan results, the programs NRecon (Bruker, Billerica, MA, USA) and DataViewer (Bruker, Billerica, MA) were used. The NRecon software was responsible for the reconstruction of the object sections. Here, the object sections for all acquired angles were fed. The software used the “Modified Feldkamp multi-slice volumetric (cone-beam) reconstruction algorithm” to generate three-dimensional data from the object slices in the form of transverse sectional images from the object sections. In this way, artifacts that occurred during the scan could already be compensated for during reconstruction [34].

The sample holder was investigated for an optimal material that would provoke the least artifacts in the scans. Polytetrafluoroethylene (PTFE) was shown to have the most promising results when considering low amounts of artifacts and low absorbances, compared to other materials. In the middle of the particle carrying pocket (ca. 165 mm³) a needle was positioned that decreased particle-particle interactions and simplified the evaluation of the scans. The bottom part could be screwed and fixed to the rotary sample plate inside the sample chamber of the µCT.

#### 2.1.2. Substance System

As a model substance system, sucrose was used due to its ease in acquisition. Two different common crystallization processes and sieve size fractions were used to demonstrate the functionality of the generated setup and workflow. The properties can be found in Table 3.

### 2.2. Applied Artificial Intelligence

To meet the requirements of this work, the artificial intelligence had to be able to differentiate between particle, sampler and background. Furthermore, for the determination of particle size distribution, the size of each particle in the distribution had to be known. This meant that, with the help of the AI-based algorithm used in this work, individual particles had to be distinguished and assigned to a unique identification. Post-processing then needed to be able to use the output of the software to reconstruct the individual particles in the bulk sample.

For the merging of individual particle images on the image slices into a complete particle, the spatial position, as well as the determination of the area of the individual images being as accurate as possible, were of crucial importance. In contrast to two-dimensional methods, such as the measurement of the particle size distribution on the basis of individual particle images, an extremely high detection rate of at least 80% of the particles within one image was required for proper three-dimensional reconstruction. This requirement resulted from the fact that a particle was represented in this procedure on many individual images, layer by layer, and reconstructed from these layers. If one or more layers of the particle were not recognized by the AI, a real particle would be divided into several non-existent individual particles, which would negatively influence the particle size and, thus, also the particle distribution. Thus, either a near-total detection had to occur, or post-processing steps had to be used to counteract this. However, post-processing could not create new data, only provide new information on the 3D structure. Therefore, enough data for interpolation or extrapolation to approximate the 3D representation was necessary. For this reason, even with solid post-processing, a high detection rate of at least 80% became necessary. This requirement profile could be met by the CNN “Mask R-CNN” (regional convolutional neural network), developed by Facebook AI Research (FAIR). Mask R-CNN is capable of object segmentation and was proved to have the highest accuracy of all neural nets submitted to the COCO Segmentation Challenge [35]. It makes use of the implementation of matterport’s Github repository Mask_RCNN. This implementation works with Python3, Keras, and Tensorflow [36]. A standard CNN works with a sliding window for object detection, so object detection can take place within the window. However, in the past this only led to unsatisfactory results with a “mean Average Precision” (mAP) of 30.5% on the VOC 2007 dataset [37]. Mask R-CNN, on the other hand, is an extension of Faster R-CNN, which, in turn, builds on Fast R-CNN or R-CNN [35,38].

The Mask R-CNN consists of a framework that can be divided into two stages. The first stage is characterized by what is eponymous with the R in its name: the Region Proposal Network (RPN). In this first stage, the image is fed into a CNN, which has the purpose of finding regions of interest (ROI) and feeding them to the next stage. The second stage consists of the classification of each ROI, as well as the generation of bounding boxes for the detected objects, and, finally, the generation of pixel accurate masks [36,38].

#### Image Labeling and Training of the Network

**Figure 2:**
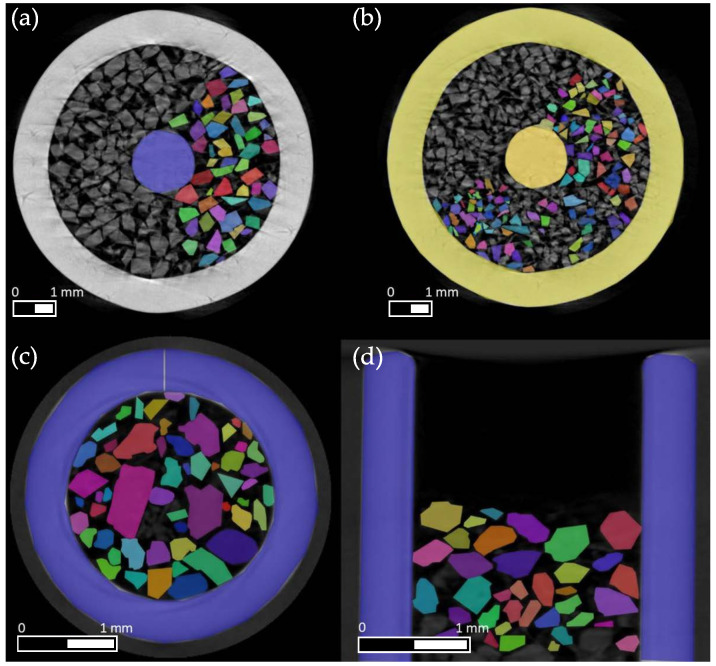
Sample of the labeled dataset of the µCT measurements of sucrose bulk samples. (**a**) shows particles with a sieve size of 250–400 µm. (**b**) shows particles with a sieve size of 180–250 µm. At the bottom, significantly larger particles from a non-sieved sample can be seen in radial (**c**) and axial directions (**d**) (without the needle in the sampler). In each of the images, a portion of the particles was manually labeled for training purposes.

The data set used in this work is described in Table 4. In total, it included 135 cross-sectional images of sugar particles, 85 images corresponding to the radial view (compare Figure 2a–c and 50 images to the axial view (compare Figure 2d).

Furthermore, different scans and particle sizes were included, and, thus, 62 images from three scans of a particle cluster of mixed size were used. For training on detection, with attention to smaller particles, sieve fractions of 180–250 µm and 250–400 µm classes were filtered and also scanned three times each. Finally, 29 images of the smaller 180–250 µm fraction and 44 images of the 250–400 µm fraction were included in the training. The number of annotations were also of significant importance because, unlike many other datasets, there might be many individual objects of the same class in the image. Thus, in the 135 images used, a total of 3400 objects were annotated, of which 90 objects corresponded to samplers and 3310 objects to particles. Moreover, data augmentation was used to further enlarge the data set by shearing and rotating it. Training Mask R-CNN on the prepared dataset required the adjustment of the parameters presented at the beginning of the subsection. The adjusted learning rate, which corresponded to the initial value of the real learning rate, was set to 0.0005. However, a higher learning rate of 0.001, which would speed up the training, resulted in stronger oscillations of the loss metrics in this work. For the decay of the learning rate by the stochastic gradient descent (SGD) procedure [39,40], a momentum of 0.9 and a decay of 0.0001 were used. These values corresponded to the default setting in the implementation of Mask R-CNN used. By splitting the dataset into 80% training and 20% validation data, the training dataset reached a size of 111 frames and the validation dataset reached a size of 28 frames. The CNN model’s predictive accuracy was assessed from the validation dataset. This set of labeled data was similar to the training dataset, but it was not used during the training step. Hence, the validation dataset could be interpreted as unseen information data by the CNN model. To compute the validation accuracy, the CNN inferred particles from the validation images were compared against the manually labeled images of the validation data set [41]. Since one image was randomly selected from the dataset with each step of an epoch, the number of steps per epoch was based on the number of images and was set to 100 images for training. The discrepancy between the number of images and steps was due to a desired random factor, so that additional random dynamics were introduced by training steps that always changed slightly. After every 50th training step, a validation step occurred. The validation served the evaluation of the training, and the informative value thereby increased with a higher number of steps. However, the computational effort also increased and, thus, the training time increased [36,38].

### 2.3. Measurement and Reconstruction

After the CNN had been trained, as described in the previous section, it was ready for use. Therefore, a scan was performed and analyzed by the AI algorithm. The data provided from the detection by the algorithm fundamentally consisted of the classification, the position in the image, and the associated masks. This trio of data was stored for each detected particle in each image, and was processed further, as described in the section Post-processing. The reconstruction and post-processing software was openly available within the scope of this work.

#### 2.3.1. Post-Processing

**Figure 3:**
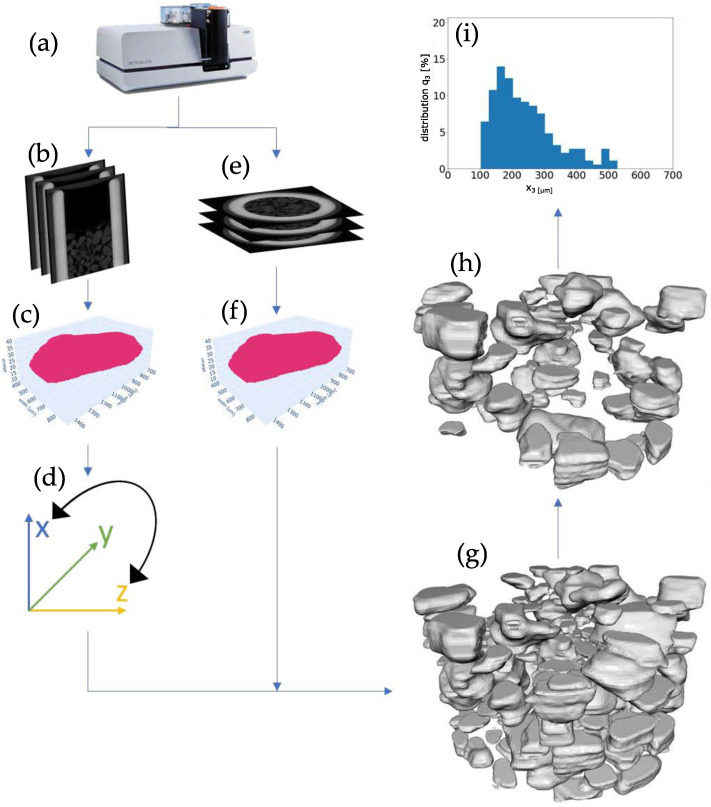
Workflow of the measurement procedure and post-processing. Samples were given to the sampler and scanned in the µCT (**a**). Scans were reconstructed into radial and axial 2d images (**b**,**e**). From the scans, the artificial intelligence was applied and point clouds in both directions were created (**c**,**f**), After transformation (**d**), the point clouds were reconstructed into a three dimensional representation (meshe) (**g**). After falsely identified particles were sorted out (**h**) the PSD was determined (**i**).

The post-processing is shown step-by-step in Figure 3. It was carried out using an Intel Xeon(R) Gold 6130 CPU @2.10 Ghz and the whole evaluation routine took 3.5 h in total, of which the AI-based evaluation took 132 min. Both the transverse images and the images transformed with ImageJ [42] to the sagittal direction, i.e., data from µCT (a), served as the source material. For performance reasons, dynamic downscaling was performed after detection. From an original resolution of 700 pixels in both spatial directions of the processed images, the data load in further post-processing was reduced by a factor of 4–16. During this process, the dimensions of the masks and regions of interest (ROI) boxes created in the detection were reduced by creating a new averaged point from every two to four points of the two spatial directions. To be able to evaluate the data, the particles had to be reconstructed by identifying and combining all the masks of a detection direction that made up a particle see Figure 3b,e. With the help of these combined masks, the volume of the individual particles could then be determined. Moreover, each particle was assigned a unique identification number (ID) in the step, which allowed for a PSD to be generated with the quantity type number, too. However, the AI-based algorithm did not work without some errors. To estimate whether a reconstruction was successful, the resulting surface was evaluated. With this, sphericity could be determined, which served as a criterion for the shape and, thus, the reconstruction of the particle. Surfaces could not be directly read from the generated masks of the particles. However, the calculation of the surface could be done using a mesh. A mesh is a surface construct of a body, usually consisting of triangles. To create this, the merged particle masks of a detection direction were converted into a point cloud ((c) and (f)), which was then hollowed out to reduce the data load. Up to this point, the data acquired in the transverse and sagittal directions were analyzed separately. The point cloud, which was generated from the axial direction, was transformed into the coordinate system of the radial direction (d) and then combined with the point cloud of the radial direction. From these point clouds, a mesh was generated by applying the Poisson algorithm for each particle (g), and the surface area was calculated from it. Then, the Wadell sphericity was determined, which was used to filter inappropriate meshes (h). From the cleaned data, and taking into account the resolution of the scan, the PSD was determined (i).

#### 2.3.2. Reconstruction

**Figure 4:**
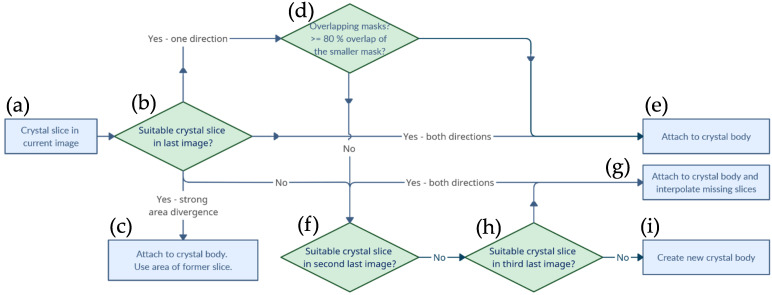
Visualization of the algorithm responsible for the reconstruction of the segments detected on each slice into individual volumes. From the particle in the current slice (**a**), the next and previous slices were looked at to determine whether a similar particle shape occurred (**b**) and if so the current particle was attached to the existing particle body (**d**,**e**) Otherwise, in the case of a strong area divergence, the contour of the previous slice was recycled (**c**) or missing slices interpolated (**g**), or it marked the beginning of a new particle (**f**,**h**,**i**).

In the following, the individual points of post-processing are discussed in more detail. For the merging of the particle cuts into whole particles, they were consecutively iterated through all but the first image, going through the scheme of Figure 4 for each particle cut in the image.

The first image served as the initial state, so each particle cut detected here was counted as the beginning of a new particle (a). For merging with subsequent cuts, the first step was to check whether a spatially similar particle cut existed for the currently considered particle cut in the previous image (b). This decision making was based on the bidirectional comparison of the bounding boxes with the center points of the bounding boxes between the cuts. Thus, it was verified both that the center of the current particle was in the bounding box of the previous one and that the center of the previous particle was in the bounding box of the current one. This provided a spatial mapping and the current particle cut was attached to an existing particle. Now, however, the mask of the current particle cut could deviate greatly; for example, due to small bumps on the particle surface, unclean segmentation of neighboring particles, or flat surfaces that were oriented approximately the same as the cut planes. Therefore, in the case of a successful bidirectional alignment, the surface was also checked. If the relative deviation between the neighboring masks was too large, the surface of the mask of the already existing particle image was used for the volume calculation (c). If the position matching was successful in only one direction, for example, if the new face was exceptionally small or large, a pixel-by-pixel overlap of the masks was calculated to assess whether the particle cut should be added to the structure (e). This happened as soon as the smaller of the two overlapping masks exhibited an overlap of at least 80% with the larger mask (d). If no matching particle structure was found in the last image section, this might indicate that the AI algorithm had mistakenly missed one or not recognized one or more particle cuts. To compensate for the detection rate, a search was started in the penultimate image slice (f). If there was a bidirectionally matching particle structure here, the particle cut was added (g). However, since a plane was now missing between the structure and the newly added particle slice, a plane was created by an interpolation of the surfaces and the center positions of the enclosing cuts. If there was also no matching particle structure in the penultimate image section, a bidirectionally matching structure was also searched for one level lower and an interpolation of the faces and center positions for both missing levels was performed (h). A further level of interpolation to the subsequent cut was not performed. If the particle cut ran through the algorithm, without finding a suitable particle structure for merging, a new particle structure was created (i). If the particle formations were now complete, the volume of each particle could be calculated by summing all voxels and multiplying by the third power of the resolution of the µCT. Voxels corresponded to the pixels of the image slices, since the distance image slices corresponded to the thickness of the resolution. After the particles were successfully assigned, a three-dimensional matrix was created with all masks of the particles. The dimensions of this matrix corresponded to the spatial direction, and, therefore, the ID of the particles was stored as a value within the matrix, so that particle “5” consisted of a collection of points with the value 5. This storage had the disadvantage that, in the case of overlapping voxels of the particles, a value was formed which was no longer related to the identification information. To avoid this, overlapping voxels were deleted from the matrix. The cleaned matrix was then used for the generation of a point cloud.

#### 2.3.3. Generation of Meshes

Since detection took place in both the axial and radial directions, two separate point clouds existed which showed different orientations. In order to bring both on one basis, a coordinate transformation and translocation of the sagittal orientation to that of the radial coordinate system was necessary. The point clouds were then combined. However, a simple summation of the two point clouds was not purposeful. Two different IDs (one for each of the directions) were available for each particle. For this reason, a fusion of the identification information had to take place. For this purpose, a cuboid was placed around each particle, which exactly enclosed the maximum dimensions of the particle. From this cuboid an equivalence diameter and a center point were formed, assigned to the ID and, thus, a volume equivalent sphere could be created for each particle. As a criterion for the fusion of the IDs, it was checked as to whether centers of formerly axial projection direction particles were located within the equivalence sphere of the transverse particles. If this was the case, the IDs of the axial particles were overwritten with those of the location-matching radials. Subsequently, all voxels of an ID were extracted from the point cloud and assigned to a function for the creation of a triangle mesh, so that, for each particle individually a mesh was generated. The mesh generation was performed using the Poisson algorithm, which could be summarized in four steps. First, via a function of the open3D toolbox in Python, the normals of the points to the surface were determined. At points close to the surface of a body, the gradient of an indicator function corresponded to the inward oriented surface normals. Thus, the oriented point samples could be used as samples of the gradient of the indicator function of the model and the indicator gradients could be generated. The gradients could then be used to determine the indicator function, which was used to finally also determine the surface [43]. The meshes were then smoothed using the Taubin algorithm. Common methods refine the number of triangles in order to represent less sharp edges. However, the volume of the solid is reduced in the process. The Taubin algorithm has the special feature that the volume of the mesh remains untouched. Since a mesh should consist of a closed surface of triangles, in this surface each triangle has three triangles as neighbors and, thus, each edge on the body belongs to two triangles. This property was used to reduce artifacts, by removing all edges which only belonged to a single triangle and, therefore, were not part of a closed surface. As a final step in the mesh generation, the mesh was checked for the surface closeness necessary for a surface to be generated from the mesh [44].

#### 2.3.4. Wadell Sphericity

**Figure 5:**
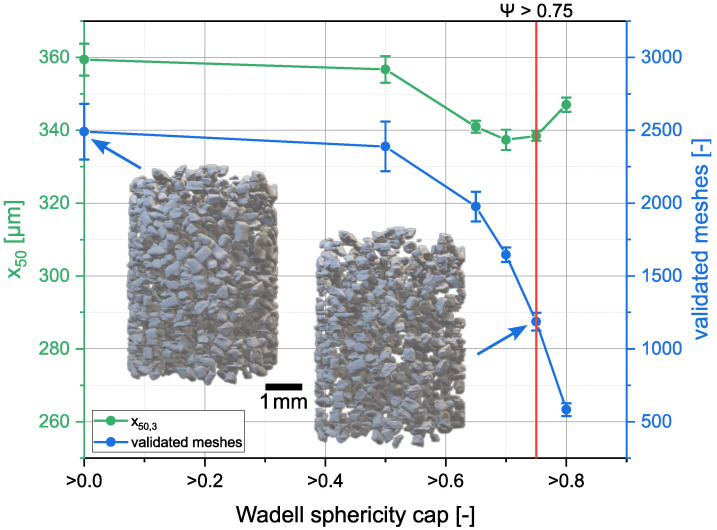
Evolution of the $x_{50,3}$ and number of validated meshes when allowing only particles above a certain Wadell sphericity. For demonstration purposes, sucrose with a sieve fraction of 250–400 µm was used. The same sample was measured three times. The sample was taken from, and put back into, the sampler between the measurements.

Finally, the Wadell sphericity with

(1) $$\psi =\sqrt[{3}]{\frac{36\cdot \pi \cdot V^{2}}{A}},$$

of the individual particles was determined with the volume and the surface of the meshes, in order to filter out incompletely, or incorrectly, recognized particles. At this point, the mesh volume was always used for the calculation of the Wadell sphericity, so that mesh volume (*V*) and mesh area (*A*) were always used for calculation. The influence of the threshold value is shown in Figure 5. Detection, reconstruction and mesh generation were performed as described above. Without filtering by means of Wadell sphericity, about 2500 individual particles were detected in the measurements. However, we observed a high number of small platelet-forming particles. These occurred when a particle was detected only sporadically, creating individual masks. Furthermore, many meshes were created which had a flat surface in the detection direction at the beginning and at the beginning of the particle. These flat areas were caused by poor detection of smaller particle cuts, so that mostly the particle tips were not detected. The detection in a second spatial direction with fusion of the masks determined for the particle intended to counteract this phenomenon. Another cause for the meshes with uncommon shape was the unclear separation of two particles in one detection direction, so that two adjacent, possibly agglomerated, particles were detected as a single particle. These erroneous particles occurred only in lower number when filtered with threshold ψ>0.65. For this reason, a further selection was made by a sphericity ψ>0.75 which let ca. 50% of the original particles remain. With a threshold value of ψ>0.8, a larger fraction of the flattened and deformed particle bodies was removed, but this drastically decreased the total number of mesh bodies and left only ca. 600 particles.

#### 2.3.5. Precision

**Figure 6:**
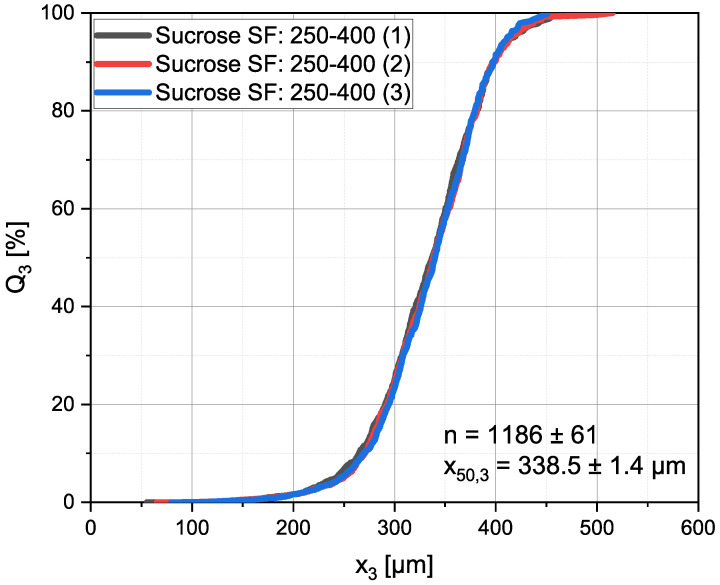
$Q_{3}$ distribution of the same sample measured and processed three times. The sample was taken from, and put back into, the sampler between the measurements. For demonstration purposes, sucrose with a sieve fraction of 250–400 µm, was used.

In Figure 6 the $Q_{3}$ distribution can be seen for a single sample of sucrose particles of the sieve fraction 250–400 µm. The sample was measured and processed, as described above, three times. Between the measurements the particles were taken from the sampler and put back into it, so that they lay in a different orientation in the bulk. From the diagram, good agreement is visible between the three data sets. $x_{50,3}$ (338.5 ± 1.4 µm) and the amount of analyzed particles (1186 ± 61) only deviated in a negligible range.

### 2.4. Analytical Reference Methods

The standard particle size measurement technologies that were used on a regular basis are introduced below. These were then used to set the results produced with the presented method into perspective.

#### 2.4.1. Sedimentation Analysis

For the sedimentation method in a time and spatially resolving spectrometer (LUMiReader^®^ PSA, LUM GmbH, Berlin, Germany) density and viscosity information was required to calculate the particle size distribution via software (SepView^®^ 6, LUM GmbH, Berlin, Germany), based on Stoke’s law. The analyzed suspension samples were created by mixing the sucrose particles of interest in ethanol due to the low solubility (0.0003 g·g^−1^) [45]. About 3 mL of the sample were introduced to a sampling tube with a height of approximately 50 mm, which also acted as maximum sinking distance during the measurements. This method was successfully used for the sieve particle size of 180–250 µm. For the larger particles the sedimentation velocity was too high and could not, therefore, be detected by the analytical equipment [46,47,48].

#### 2.4.2. Laser Light Diffraction

Laser light diffraction works by exploiting the relationship between light scattering, its angle and intensity, and particle size. The larger the particle, the smaller the angle and, thus, the higher the intensity of the scattering. Particle sizes were measured via laser light diffraction (Mastersizer 3000, Malvern Instruments, Malvern, UK) in a wet dispersion unit (Hydro SV).

#### 2.4.3. Image Analysis

As a comparison, the sieved particles were optically investigated using the segmentation tool Sefexa [49] for image analysis. Here, particles could be investigated non-invasively without large equipment. A microscope was equipped with a camera. The particles of interest were placed on a microscopic glass slide and photographed. From the manually determined projection area of the individual particles, the $Q_{2}$ distribution was converted into the volumetric $Q_{3}$ distribution, by using the circular area equivalent diameter. For each experimental investigation at least 1000 particles were evaluated. In order to achieve this number, 24 images for the particle sieve size of 180–250 µm, and 53 images for the particle sieve size of 250–400 µm, were evaluated.

## 3. Measurement of Sucrose Particles

**Figure 7:**
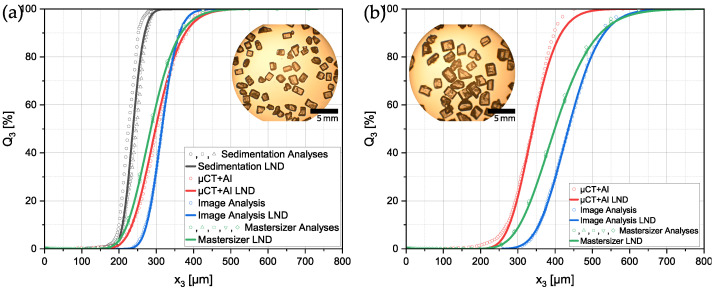
Volumetric particle size distributions measured with different strategies of the sieve size fractions: (**a**) Sucrose 180–250 µm, (**b**) Sucrose 250–400 µm. A lognormal distribution (LND) was fitted to each of the raw datasets.

In the following, the results of the different previously described methods are demonstrated. Figure 7 shows the volumetric PSDs of the two sieve fractions of the sucrose particles. Despite the different measurement methods, the respective necessary assumptions enabled the plotting of all datasets in a single diagram for comparison.

In Figure 7a the PSDs of the smaller sieve size fraction of the sucrose particles are shown. $x_{50,3}$ values range from 237.5 µm for the sedimentation analysis to 315.08 µm of the manual image analysis. There was a maximum difference of 77.58 µm for the $x_{50,3}$ across the methods. The fact that particles were settling faster in the measurement tube during the sedimentation analysis explained why smaller particles that needed more time for settling were more prone to detection by the analysis system. This, in turn, could be the reason why the sedimentation analyses detected a PSD with a smaller $x_{50,3}$. The other three methods were fairly close to each other regarding the $x_{50,3}$ with the image analysis yielding the biggest particles ($x_{50,3}$).

For the results shown in Figure 7b, it was not possible to add the sedimentation analysis due to the larger size and, therefore, bigger sinking velocity in ethanol of the particles. The determined PSDs had a higher discrepancy than that for the smaller particles. The $x_{50,3}$ ranged from 339.51 µm up to 435.51 µm. It was noted that the presented measurement method seemed to yield the most accurate results, when only taking into account the sieve size of the particles. Contrarily, the image analysis yielded a distribution with an $x_{50,3}$ larger than expected. This could be explained in two different ways. Firstly, for optical anaylsis methods it is common that smaller particles are under-represented simply because of their size. The detection can be hard for the operator and they tend to be covered up by other bigger particles in the image. Secondly, in image analysis, the particles usually have a preferred orientation during the capture of the image. In this case, particles positioned on a microscopic glass slide usually lie on their largest face which could cause a systematic error in the measured PSDs. This was due to the fact that, from the projection area, the volume of a sphere was calculated and used for the volumetric distribution.

In particular, for the comparison of PSDs in literature, it is common practice to only compare measurement results done with the same strategy. A comparison across different methods is not trivial, because different assumptions and approximations lead to different systematic errors. From the results obtained, it could be concluded that sedimentation analysis had the tendency to systematically lead to diameters that were too small. This might have been due to the fact that a spherical shape was assumed by the evaluation software. Since the particles differed from that idealized shape a systematic error occurred. On the other hand, image analysis using a microscope seemed to have a systematic error leading to too large diameters. Since particles were not ideally shaped, in images a certain orientation of the particles of interest was over-represented. In the present case, the particles usually rested on their largest face and, therefore, presented their largest projection area to the observer, resulting in possibly shifted PSD results. Additionally, the borders could be artificially enlarged with different light and contrast settings.

Since, in the presented method, a criterion with the Wadell sphericity was implemented to filter out incorrectly reconstructed meshes that did not comply with particle specifications, it was expected that, for substance systems with distinct particle shapes, the criterion would need to be adjusted accordingly. Furthermore, as the reconstruction method relied on scanning and reconstruction of slices to volume bodies, the method might be especially prone to errors for plate forming substance systems. For substance systems with particle shapes that were close to a sphere (high Wasell sphericity) the method was expected to perform more precisely. As sucrose grows particles that are monoklin–sphenoidic this was well represented here [50].

## 4. Conclusions and Outlook

An evaluation method for identifying particle size, PSD and shape, using µ-CT and Artificial Intelligence for image analysis, was developed and compared to other measurement techniques, such as sedimentation analysis, laser light diffraction, and microscopic image analysis. From a bulk sample it was possible to identify individual particle characteristics.

In contrast to conventional measurement, the three-dimensional evaluation using the µ-CT seemed to overcome hurdles as it considered the particles’ true shapes. The AI algorithms instance segmenation exhibited high accuracy in detecting 80% particles shapes, and the particles were reconstructed to their true shapes with a resolution of down to 8 µm voxels. Here, the determined shape was used to calculate the Wadell sphericity of individual particles which fell short of its possibilities. Degree of agglomeration, particle shape distributions, and polymorphisms could be investigated with an accuracy of 8 µm.

As this contribution is a demonstration of the measurement principle and the applied methods to determine the CSD of a bulk sample, the feasibility, based on preliminary results, was successfully demonstrated using sucrose as the model substance. Using commonly used and established analytical methods the results were validated. Future demonstrations will include the application of the method to further substance systems to show its versatility.

## Figures and Tables

**Table 1:** Common techniques to measure the particle size distribution in crystalline products.

Method	Range	Measured Quantity—Symbol	Description	Comments	Ref.
Sieving	20 µm–125 mm	$d_{a}$	Width of the minimum square aperture through which the particle will pass	+ easy + cheap − offline	[22,23,24,25,27,28]
Sedimentation	0.01 µm–50 µm	$d_{St}$	Free-falling diameter in the laminar flow region	+ cheap − low range − offline	[22,23,25,26,27,28]
Optical microscopy	0.5 µm–500 µm	$d_{p}$	Diameter of a circle having the same projected area as the particle in random orientation	+ shape/size − low number of particles − offline	[22,23,25,26,27,28]
Laser light diffraction	1 µm–1000 µm	$d_{c}$	Particle chord length	+ powders and suspensions + offline and online − expensive	[23,25,26,27,28]
Focused beam reflection measurement	0.5 µm–1000 µm	$d_{c}$	Random particle chord length	+ online − expensive	[25]
Micro-computed tomography	10 µm–1000 µm	$d_{V}$	Diameter of a sphere with the same volume	+ precise + actual shape − expensive − offline	This work

**Table 2:** Parameters for the here presented scans.

Parameter	Value
Radiation source voltage	40 kV
Radiation source current	200 mA
Exposure time	55 ms
Spatial resolution	8 µm
Angle step size	0.12°
Shots per angular step	5
Scan duration	44.5 min

**Table 3:** Evaluated particle species and sieve size fractions.

Species	Sieve Size Fraction	Supplier
Sucrose	180–250 µm 250–400 µm	Südzucker AG, Mannheim, Germany

**Table 4:** Training data set of Sucrose particle species and their amount and sieve size fractions.

Training Data	Sieve Size Fraction	Number of Images [-]
Sucrose three scans of a particle cluster of mixed size	180–400 µm	62
Sucrose small	180–250 µm	29
Sucrose large	250–400 µm	44

## Data Availability

The Code can be found here: https://tu-dortmund.sciebo.de/s/uDX9wd3IlSPDZ0D (accessed and uploaded on 15 December 2022).

## Abbreviations

The following abbreviations are used in this manuscript:

AI	Artificial Intelligence
CNN	Convolutional Neural Network
COCO	Common Objects in Context File Format
$d_{i}$	diameter/length
ID	Identification Number
LND	Log Normal Distribution
mAP	Mean Average Precision
n	Amount
PLA	Polylactic Acid
PSD	Particle Size Distribution
PTFE	Polytetrafluoroethylene
ψ	Wadell Sphericity
$Q_{3}$	Cummulative Volumetric Distribution
AI	Artificial Intelligence
ROI	Region of Interest
RPN	Region Proposal Network
SGD	Stochastic gradient descent
µCT	Micro Computed Tomography
$x_{50,3}$	Median of Volumetric Distribution
